# Supplementary material for: Enzyme I facilitates reverse flux from pyruvate to phosphoenolpyruvate in Escherichia coli
Source: Nat Commun. 2017 Jan 27;8:14316. doi: 10.1038/ncomms14316 (PMC5290146; doi:10.1038/ncomms14316)
Supplement: Supplementary Information — Supplementary Figures, Supplementary Table and Supplementary Methods [file ncomms14316-s1.pdf]

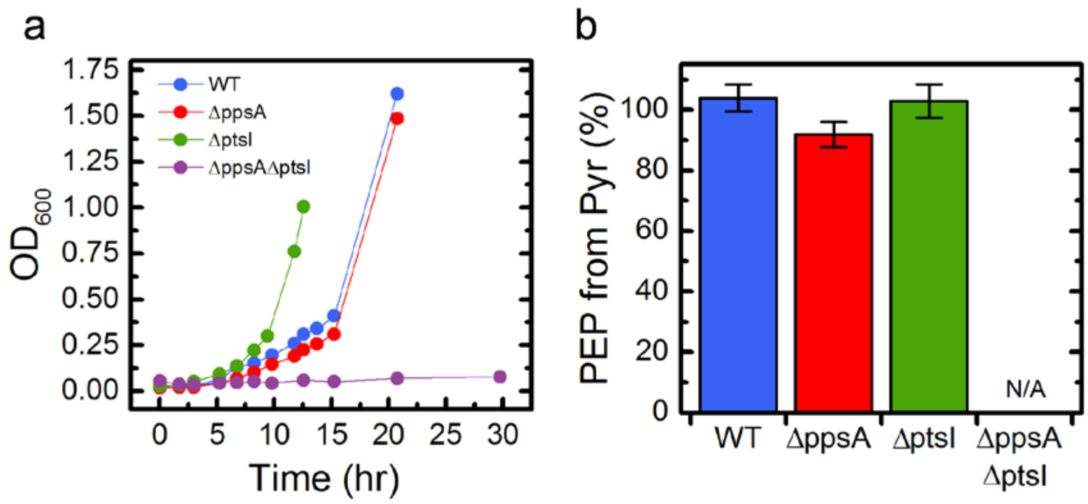

Supplementary Figure 1. (a) Growth profiles of four *E. coli* strains during growth on pyruvate: wild-type (WT), Δ*ppsA*, Δ*ptsI*, and the double knockout Δ*ppsA*Δ*ptsI*. The double knockout strain Δ*ppsA*Δ*ptsI* did not grow on pyruvate. **(b)** [1-^{13}C]alanine experiments were performed and the percentage of PEP derived from pyruvate was determined. For the three strains able to grow on pyruvate, nearly 100% of PEP was derived from pyruvate. The errors reflect propagation of GC-MS measurement errors through the calculation.

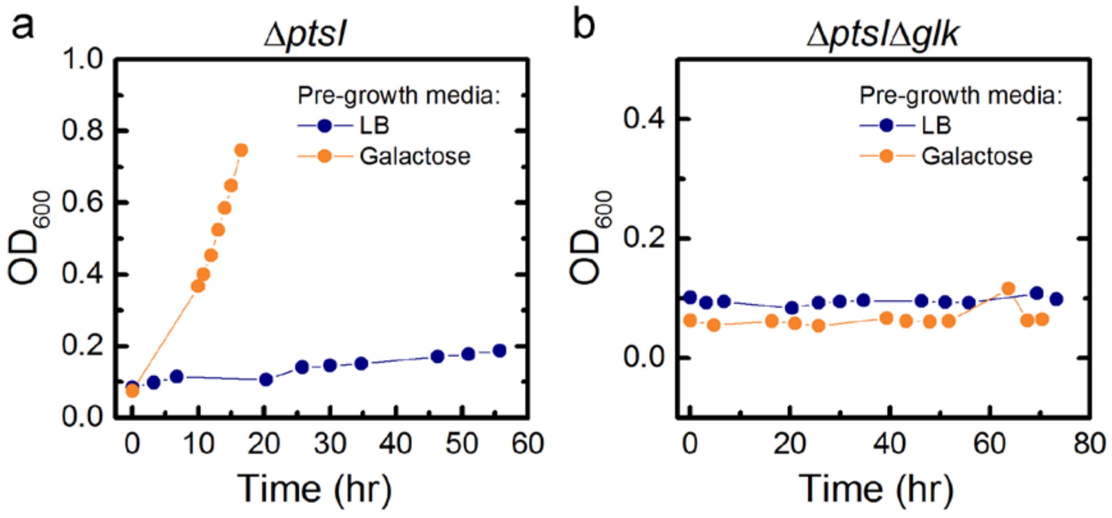

Supplementary Figure 2. (a) Growth profiles of the Δ*ptsI* strain on glucose. When Δ*ptsI* was pre-grown on LB medium, little or no growth on glucose was observed. When Δ*ptsI* was pre-grown on M9 medium with galactose, the cells grew immediately on glucose without any lag phase. **(b)** The double knockout Δ*ptsI*Δ*glk* did not grow on glucose, regardless if the cells were pre-grown on LB medium or M9 medium with galactose. These results demonstrate that PTS transport is inactive in the Δ*ptsI* strain, but instead glucokinase (*glk*) is responsible for glucose phosphorylation.

Supplementary Table 1. *E. coli* knockout strains from the Keio knockout collection used in this study

Knockout Gene	Plate-Row-Col ID	JW_id-Strain
ppsA	3-A-3	JW1692-1
ptsG	55-G-3	JW1087-2
crr	57-H-8	JW2410-1
ptsH	57-F-8	JW2408-2
ptsI	57-G-8	JW2409-1

Supplementary Methods.

Calculating the percentage of PEP derived from pyruvate

Correction of labeling data for unlabeled biomass (after natural abundance correction)

Since we are measuring labeling of amino acids from hydrolyzed biomass proteins, the mass isotopomer data must be corrected for unlabeled biomass that was present prior to the introduction of ^{13}C-alanine. The fraction of *old unlabeled* biomass and fraction of *new* biomass (i.e. generated after the introduction of ^{13}C-alanine) is calculated as follows:

Fraction of *old unlabeled* biomass = Ala_{M0}

Fraction of *new* biomass = $1 - Ala_{M0}$

The mass isotopomer data of other amino acids, e.g. valine, phenylalanine, aspartate, can be corrected for the presence of *old unlabeled* biomass as follows:

$$Val_{M0}^{corr} = \frac{Val_{M0} - Ala_{M0}}{1 - Ala_{M0}} \qquad \text{(for M0)}$$

$$Val_{Mi}^{corr} = \frac{Val_{Mi}}{1 - Ala_{M0}} \qquad \text{(for M}i\text{, } i > 0\text{)}$$

Tracer experiments with [1-^{13}C]alanine (growth on acetate and pyruvate)

For [1-^{13}C]alanine tracer experiments, the fraction of PEP derived from pyruvate is calculated from the M1 labeling of pyruvate and M1 labeling of PEP. Labeling of pyruvate is inferred from that of valine. GC-MS analysis of valine produces *m/z* 288 fragment which contains carbons C1-C5 of valine. C1 of valine is derived from C1 of pyruvate, and the remaining carbons are derived from C2-C3 of pyruvate. Since carbons C2-C3 of pyruvate are unlabeled, the M1 labeling of *m/z* 288 fragment reflects the M1 labeling of pyruvate:

$$Pyr_{M1} = Val288_{M1}^{corr}$$

The M1 labeling of PEP is inferred from the M1 labeling of *m/z* 302 fragment of phenylalanine, which contains C1-C2 of phenylalanine that are derived from C1-C2 of PEP:

$$PEP_{M1} = Phe302_{M1}^{corr}$$

Thus, the fraction of PEP derived from pyruvate is calculated as follows:

$$\%PEP\ from\ Pyr = \frac{PEP_{M1}}{Pyr_{M1}} = \frac{Phe302_{M1}^{corr}}{Val288_{M1}^{corr}} = \frac{Phe302_{M1}/(1 - Ala260_{M0})}{Val288_{M1}/(1 - Ala260_{M0})} = \frac{Phe302_{M1}}{Val288_{M1}}$$

In the above equation, we have assumed that the contribution of oxaloacetate to PEP_{M1} is minimal, which we have confirmed by measuring aspartate labeling directly and showing that it was negligible in all cases.

Tracer experiments with [U-^{13}C]alanine (growth on glucose and xylose)

For [U-^{13}C]alanine tracer experiments, the fraction of PEP derived from pyruvate is calculated by least squares regression using the measured mass isotopomer distributions (MID) of PEP (determined from phenylalanine *m/z* 302 fragment, C1-C2), oxaloacetate (OAC) (determined from phenylalanine *m/z* 302 fragment, C1-C2), and PYR (determined from valine *m/z* 260 fragment, C2-C5), using the following equation:

$$MID_{PEP,C1-C2} = (\%PEP\ from\ Pyr) * MID_{Pyr,C1-C2} + (\%PEP\ from\ OAC) * MID_{OAC,C1-C2} +$$

$$(\%PEP\ from\ gluc\ or\ xyl) * MID_{unlabeled}$$

The mass isotopomer distribution of pyruvate (carbon atoms C1-C2) is determined as follows:

$$MID_{Pyr,C1-C2} = \begin{bmatrix} Pyr_{C1-C2,M0} \\ Pyr_{C1-C2,M1} \\ Pyr_{C1-C2,M2} \end{bmatrix} = \begin{bmatrix} \sqrt{Val260_{M0}^{corr}} \\ 0 \\ 1 - \sqrt{Val260_{M0}^{corr}} \end{bmatrix}$$

The mass isotopomer distributions of PEP and OAC (carbon atoms C1-C2) are determined as follows:

$$MID_{PEP,C1-C2} = \begin{bmatrix} PEP_{C1-C2,M0} \\ PEP_{C1-C2,M1} \\ PEP_{C1-C2,M2} \end{bmatrix} = \begin{bmatrix} Phe302_{M0}^{corr} \\ Phe302_{M1}^{corr} \\ Phe302_{M2}^{corr} \end{bmatrix}$$

$$MID_{OAC,C1-C2} = \begin{bmatrix} OAC_{C1-C2,M0} \\ OAC_{C1-C2,M1} \\ OAC_{C1-C2,M2} \end{bmatrix} = \begin{bmatrix} Asp302_{M0}^{corr} \\ Asp302_{M1}^{corr} \\ Asp302_{M2}^{corr} \end{bmatrix}$$

Finally:

$$MID_{unlabeled} = \begin{bmatrix} M0 \\ M1 \\ M2 \end{bmatrix} = \begin{bmatrix} 1 \\ 0 \\ 0 \end{bmatrix}$$
